# Cloning and phylogenetic analyses of serine/threonine kinase class defense-related genes in a wild fruit crop 'chestnut rose'

**DOI:** 10.1186/1756-0500-3-202

**Published:** 2010-07-18

**Authors:** Qiang Xu, Xiuxin Deng

**Affiliations:** 1 National Key Laboratory of Crop Genetic Improvement, Huazhong Agricultural University, Wuhan 430070, Hubei, PR China

## Abstract

**Background:**

Chestnut rose (*Rosa roxburghii *Tratt) is a promising wild fruit crop in Southwest China. However, chestnut rose suffers from several important diseases such as powdery mildew and black spot. Cloning and phylogenetic analysis of plant immunity related genes will strengthen the evolutionary knowledge of plant immune system and will facilitate the utilization of candidate genes in disease resistance breeding programs.

**Findings:**

Serine/threonine kinase (STK) genes, encoding one of the important proteins for defense signal transduction, were cloned from 'chestnut rose'. Fifteen STK sequences were obtained by degenerate PCR. Sequence analysis showed that nine of them have continued open reading frames, and they are separated into five classes based on sequence analysis. Interestingly, one of the classes (STK V) showed less than 40% similarity to any other class, possibly representing new type genes from chestnut rose. Southern blotting analysis revealed that the new type STK V genes are single copy, while all the other genes have several copies in the genome. Phylogenetic analysis of STK genes from chestnut rose and 21 plant species revealed that most chestnut rose genes show close relationship with Rosaceae homologs, while the STK V genes are rather ancient and form a unique clade distantly from plant homologs.

**Conclusions:**

We cloned nine STK genes from a wild fruit crop 'chestnut rose', of which a new type of *STK *genes was identified. The new type STK genes exist as single copies in the genome, and they are phylogenetically distant to plant homologs. The polymorphic STK genes, combined with other plant immunity genes, provide plenty of resources to be utilized to defend against pathogens attack.

## Background

Chestnut rose (*Rosa roxburghii *Tratt), a wild fruit crop in Southwest China, belongs to Rosaceae family Rosa genus. It has recently been labeled as one of the three promising new fruit crops in China due to its fruit having high content of vitamin C (2000-3000 mg/100 g FW), displaying high levels of superoxide dismutase (SOD) activity, and therefore is believed to have senescence-retarding and cancer-preventing effects [1]. Unfortunately, chestnut rose crops are suffering from powdery mildew disease. After decades of breeding, an indigenous cultivar from Guizhou province of China, Guinong No. 6, was discovered to be highly resistant to powdery mildew [2]. This material provides an opportunity to investigate the mechanism underlying the generation of the powdery mildew resistance specificity in chestnut rose.

Plants rely on their innate immune system to defend against pathogens. The immune system is composed of three important steps, i.e. pathogen detection, signal transduction, and defense response initiation [3-5]. The pathogen detection protein, encoded by resistance (*R*) genes, can recognize pathogen-produced effectors and will produce a defense signal; the transmission and amplification of defense a signal requires kinase genes in the plant cell; finally the host initiates transcription of the defense-response gene including the pathogenesis-related (*PR*) gene that can confer local or systemic resistance [4,6].

Among the component genes of plant immune system, most researches are focusing on *R *genes [7]. Many *R *genes have now been cloned from a wide variety of plant species, of which the largest class is belonging to the nucleotide-binding site--leucine-rich repeats (NBS-LRR) family [7,8]. The NBS domain is highly conserved across plant species, this characteristic facilitated the cloning of large number of sequences homologous to R genes, and so-called "R gene candidates (RGCs)", from many plant species by degenerate PCR strategy. Comprehensive analysis of plant RGCs have revealed that several important molecular events including duplication, recombination, mutation, strong balancing selection, diversifying selection, and meiotic instability occur on plant *R *genes [9-11]. Compared with *R *genes, research on other component genes of the plant immune system were rather limited.

Serine/threonine protein kinase (STK) is one of the important proteins responsible for defense signal transduction. The STK domain is the major constituent of the tomato *Pto *gene which not only interacts with the avirulence proteins from *Pseudomonas syringae*, but also functions as a signal transduction mediator [12]. The STK domain is also contained in the rice *Xa21 *gene which confers resistance to *Xanthomonas oryzae *pv oryzae [13]. STK domains are conserved across plant species, and have been isolated in several plant species by degenerate PCR [14-16]. Genome-level analysis of kinase genes were mainly carried out in Arabidopsis and rice [17-21]; and evolutionary analysis revealed that ancient diversification and rapid evolution occur on STK genes [22-24].

A great effort was taken to understand what happens to the immune system in parallel with the generation of the resistance specificity in the chestnut rose powdery mildew resistance genotype Guinong No.6 in our Lab. A total of 38 and 37 NBS-type resistance gene candidates (RGCs) were cloned from the resistant and susceptible genotype, respectively. Comparative analysis showed that the RGCs are under rapid evolution and are polymorphic between resistant and susceptible genotypes [25]. Moreover, genes corresponding to the defense response including *pathogenesis related 2 *(*PR2*) and *PR5 *gene families were cloned; sequence polymorphisms including single nucleotide differences and small insertion/deletions (InDel) were observed in these plant immunity genes. Through analysis of these two kinds of plant immunity genes, we discovered some information on the chestnut rose immune system. However, knowledge on the defense-related kinase genes is lacking. This prompted us to clone the STK kinase genes and investigate their sequence features, genomic status, and evolutionary characteristics in the chestnut rose powdery mildew resistance genotype.

## Methods

### Plant materials

Guinong No.6, a powdery mildew-resistant cultivar was used for STK genes cloning. Genomic DNA was extracted from 3 g samples of young leaves using a CTAB protocol from our Lab [26].

### Isolation of STK genes

Degenerate primers were designed according to Di Gaspero and Cipriani [15] and Vallad et al., [14] targeted to isolate Serine/threonine kinase (STK) genes. Their sequences were: P3, TNGGNSANGGNGKNTTYGG; P8, AARYCNBMIAAYRTNCTICTNGAY; P2R, ACNCCRAANGARTANACRTC; P8R, TCNGGKCIAKRTANCCNAKNGTNCC. PCRs were performed on a PTC-200 thermal cycler in a total volume of 50 μl consisting of 50 ng DNA, 5 μl 10× buffer (Promega), 5 μl 10 mM dNTP, 4 μl of 25 mM MgCl_2_, 2 μl of 10 μM primers and 2 units of *Taq *polymerase (Promega). The cycling conditions were as follows: 94°C 3 min followed by 32 cycles of 94°C 1 min, 55°C 1 min, 72°C 2 min. PCR products corresponding to the expected size were excised from the gel and purified using a gel extraction column (Omega BioTek, USA). The obtained DNA fragments were cloned into pMD18-T vector (Takara Bio Inc.). Recombinant plasmid DNA was extracted by alkaline lysis. Each clone was re-amplified with M13 universal primers and then subject to restriction analysis using three restriction endonucleases (*Taq*I, *Hae*III, and *Hin*fI). Based on the restriction patterns, representative clones of each type were used for sequencing with the Bigdye Terminator V3.1 cycle sequencing kit on an ABI 3730 sequencer.

### Southern blotting analysis

Southern blotting was performed according to Xu et al., [26]. Briefly, 10 μgenomic DNA of *Rosa roxburghii *cv. Guinong No.6 and *R. roxburghii *cv. Guinong No.5 were digested with three different restriction endonucleases (i.e. *Hin*dIII, *Bam*HI, and *Taq*I). Digests were separated by electrophoresis in a 0.8% (w/v) agarose gel and transferred to Hybond-N+ membrane (Amersham). Hybridization was done at 65°C overnight. The stringency wash conditions were as follows: twice in 2 × SSC/0.5% SDS for 5 min at room temperature and once in 0.1 × SSC/0.1% SDS for 20 min at 65°C. Membranes were exposed to x-ray film at -80°C for 2-6 d.

### Phylogenetic tree construction and tree similarity analysis

Phylogenetic analyses were carried out according to Xu et al., [25]. Sequences from other plant species were downloaded from GenBank, and the detailed information on the representative genes can be viewed in Table 1. Sequences were aligned using CLUSTALX and manually edited in GENEDOC http://www.psc.edu/biomed/genedoc/. Neighbor-joining trees using Kimura's two-parameter model and maximum parsimony phylogenetic trees were constructed and bootstrap number were calculated by heuristic search in PAUP* 4.0 (Sinauer Associates, Sunderland, MA, USA). The trees were visualized using the program TREEVIEW http://taxonomy.zoology.gla.ac.uk/rod/treeview.html.

**Table 1:** Chestnut rose serine/threonine kinase (STK) gene homologs in different plant species^a^

Source	STK Protein^b^	Identity (Similarity, %)	e-value
*Arabidopsis thaliana*	NP_198220 CAB62020	71 (82)	3e-70
*Brassica rapa*	AAZ66951	67 (79)	3e-64
*Capsicum chinense*	AAQ82660 AAQ82661	69 (80)	6e-71
*Carica papaya*	AAU81602	71 (81)	6e-68
*Catharanthus roseus*	CAA97692	68 (80)	2e-70
*Cucumis melo*	AAL83882	69 (77)	3e-68
*Fragaria chiloensis*	ACA05210 ACA05220 ACA05215	69 (79)	1e-69
*Lycopersicon pimpinellifolium*	A49332	64 (76)	2e-55
*Mangifera indica*	AAT94935	67 (78)	1e-67
*Marchantia polymorpha*	BAF79940	71 (80)	9e-73
*Medicago truncatula*	ABE93789	72 (81)	4e-76
*Musa acuminata*	ABR68648	65 (74)	3e-67
*Oryza sativa*	XP_476621 EAZ36867	68 (80)	2e-72
*Phaseolus vulgaris*	AAK52027	60 (74)	1e-69
*Physcomitrella patens*	XP_001760779	72 (82)	4e-71
*Potentilla tucumanensis*	ACA05211	66 (79)	3e-67
*Platanus × acerifolia*	ACI05941	68 (78)	4e-68
*Prunus avium*	ABV30718	64 (76)	6e-82
*Solanum lycopersicum*	AAL17825	63 (75)	2e-55
*Triticum aestivum*	AAL51075	69 (78)	3e-70
*Vitis vinifera*	CAN60912	79 (87)	6e-80

^a^STK1 gene from chestnut rose was used for BLAST analysis.

^b^Representative proteins chose from the STK proteins.

## Results

### The cloning of serine/threonine kinase genes

**Figure 1:**
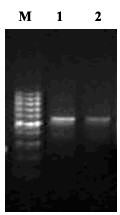
**The PCR amplification of *STK *genes from chestnut rose**. The figure showed two samples: Lane 1, *Rosa roxburghii *cv. Guinong No.6 which is resistant to powdery mildew disease; and Lane 2, *R. roxburghii *cv. Guinong No.5 which is susceptible to powdery mildew disease.

**Figure 2:**
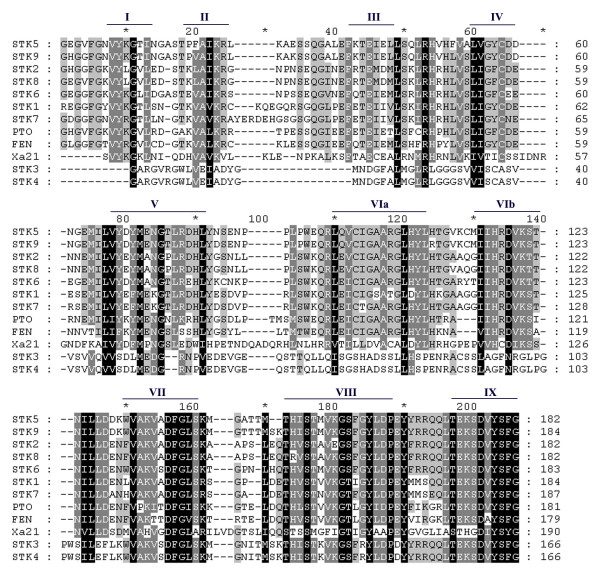
**Amino-acid alignment of chenstnut rose *STK *genes and *PTO*, *Fen*, and *Xa21 *genes**. The conserved motifs were indicated with lines according to Vallad et al., [14]. Identical amino acids are shaded in black and conservative substitutions are shaded in grey.

With degenerate primer P3 and P2R pair, a band of the predicted size (~550 bp) was observed after PCR amplification with the DNA of powdery mildew resistant genotype (Guinong No.6) as a template (Fig. 1). These expected fragments were excised from agarose gel and cloned into the plasmid vector pMD18-T. A total of 170 positive clones were picked and used for screening by endonuclease restriction pattern. Thirty unique clones were chosen for DNA sequencing, of which 15 clones contained the primer sites. BLAST analysis against GenBank database revealed that the 15 genes are highly homologous to serine/threonine kinase (STK) genes (Table 1). Conceptual translations of the above 15 sequences revealed the presence of premature stop codons in six clones. These sequences with in-frame stop codons are regarded as pseudogenes. The remained nine sequences (named as STK1-STK9 under Genbank accession number AY583621-AY583629) were regarded as STK genes by the presence of continue open reading frame (ORF) and by characteristic motifs such as VYKGVL and DVYSFG (Fig. 2).

For these nine sequences, nucleotide identity among each clone pair was determined. The identity ranged from 34-99%, with the highest occurred between STK3 and STK4. The similarity of the deduced amino-acid of these nine sequences was also calculated (Table 2). Clones with greater than 85% similarity were grouped into a single class, and this resulted the nine sequences into five classes (designated as STK-I, STK-II, STK-III, STK-IV and STK-V). Amino-acid similarity among the classes ranged from 26% (STKI vs STKV) to 79% (STKII vs STKIV).

**Table 2:** Percentage of amino-acid similarities among chestnut rose *STK *genes and the tomato *Pto *gene.

	STK1	STK2	STK3	STK4	STK5	STK6	STK7	STK8	STK9
STK2	68								
STK3	26	28							
STK4	26	28	100						
STK5	62	67	32	32					
STK6	68	79	31	31	72				
STK7	85	68	27	27	63	66			
STK8	68	97	27	27	69	78	67		
STK9	63	69	34	34	94	73	64	68	
Pto	63	67	34	24	60	65	63	67	61

It is surprising that STKV class (including STK3 and STK4 clones) showed less than 40% similarity to any other class. BLAST analysis of the STK3 and STK4 clones against GenBank database revealed that they are homologous to STK kinase gene with E-values < e × 10^-21^, also the primer sites were contained in the two clones, assuring that they are STK kinase genes. The low similarity may suggest that STKV class is a new type of STK genes in chestnut rose genome.

### Southern blotting of chestnut rose *STK *genes

**Figure 3:**
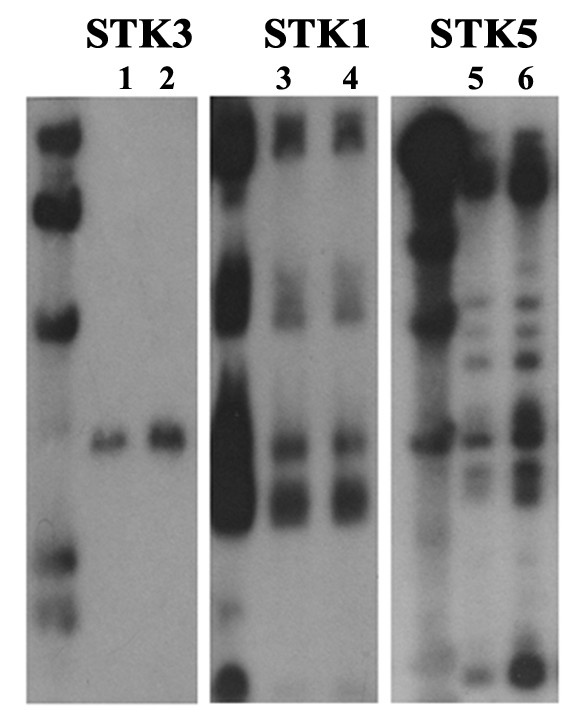
**Southern analysis of chestnut rose *STK *genes**. Lane 1, 3, 5: *Rosa roxburghii *cv. Guinong No.6 which is resistant to powdery mildew disease; Lane 2, 4, 6: *R. roxburghii *cv. Guinong No.5 which is susceptible to powdery mildew disease. The DNAs were digested with *Hin*dIII. DNA size markers (ë/*Hin*dIII) are indicated to the left of the image.

Southern analysis of the chestnut rose *STK *genes revealed that the copy number of different genes varied greatly (Fig. 3). A number of copies were observed for STK1 and STK5 genes; while STK3, the new type of *STK *gene in chestnut rose genome, only had a single copy in the chestnut rose genome. Furthermore, no polymorphisms were detected between the powdery mildew resistant/susceptible genotypes using 3 probed genes (STK1, STK3, and STK5), as revealed by Fig. 3. This data indicated that different *STK *genes have different hybridization pattern but each *STK *gene was conserved among chestnut rose genotypes.

### Phylogenetic profile of chestnut rose *STK *genes

**Figure 4:**
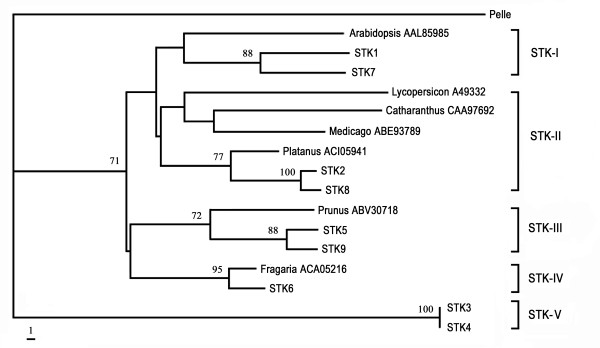
**Phylogenetic relationship of chestnut rose *STK *genes**. The trees were constructed by the neighbor-joining method by PAUP* 4.0 software. Bootstrap values (1000 replicates) with only values > 50% were shown on the branches.

Phylogenetic analysis was carried out using PAUP software according to Xu et al., [25]. The phylogenetic tree showed that chestnut rose nine *STK *genes were separated into five distinct clades (STKI-STKV), consistent with the similarity based amino-acid sequence analysis as described previously (Fig. 4).

**Figure 5:**
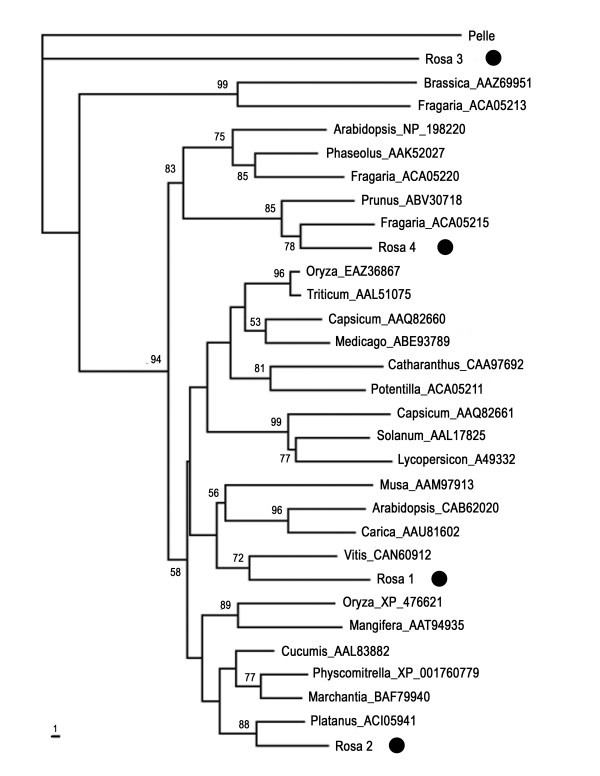
**Phylogenetic relationship of 30 plant *STK *genes (4 from chestnut rose and 26 from other plant species)**. The *STK *genes from other plant species were based on the following strategy: amino acid sequences of chestnut rose STK genes were used as a query in BLASTp searches against GenBank database, the produced top 100 sequences were used for filter out highly homologous sequences (> 85% identity) within a plant species. The remained sequences were used for phylogenetic analysis. Drosiphila *Pelle *gene was used as outgroup in this study. Rosa 1 comprises STK1 and STK7 genes; Rosa 2 comprises STK2 and STK8 genes; Rosa3 comprises STK3 and STK4 genes; Rosa4 comprises STK5, STK6 and STK9 genes. The trees were constructed by the neighbor-joining method by PAUP* 4.0 software. Bootstrap values (1000 replicates) with only values > 50% were shown on the branches.

Evolutionary relationship among chestnut rose *STK *genes and other known *STK *genes was further evaluated by phylogenetic analysis. Amino acid sequences of chestnut rose STK genes were used as a query in BLASTp searches against GenBank database for possible homologues in other plants species. The top 100 sequences were selected, and highly homologous sequences (> 85% identity) within a plant species were used to choose a representative sequence for further analysis. A total of 30 *STK *genes from 21 plant species were used in this study. The phylogenetic tree indicated that chestnut rose *STK *genes distributed in four clades; one was near to *Platanus × acerifolia *homolog, the second was neighbor to *Vitis vinifera*, the third was neighbor to *Fragaria*, and the final formed a distinct clade far from plant species but closely related with drosophila homolog gene (Fig. 5). These data suggested that the *STK *genes in chestnut rose are ancient with multi-origins.

## Discussion

Defense against pathogens involves three important steps: pathogen detection, signal transduction, and initiation defense response [4]. Most of the previous molecular studies focus on the pathogen detection *R *gene [10,11,27]. In this study, we characterized *STK *genes which encode kinase proteins responsible for the signal transduction during defense response. The genes from the relative wild fruit tree 'chestnut rose' showed close phylogenetic relationship to those from Rosaceae homologs after phylogenetic analysis of the STK genes from 21 plant species; however, some genes are a new type from chestnut rose. For example, STK3 and STK4 formed a unique clade which cannot be clustered with homolog genes from plants (Fig. 5). This indicates that from this wild fruit tree most of the *STK *genes are ancient and conserved, but with few plant-specific genes.

### The *STK *genes from a wild fruit crop 'chestnut rose'

A total of 15 STK sequences were obtained from chestnut rose, of them nine have continue open reading frame. The nine genes have been separated into five classes based on both sequence similarity and phylogenetic analyses. Four of the five classes were belonging to known *STK *kinases, while the remained one class (STKV) represents a new type. The STK3 and STK4 genes (member of STKV class) showed less than 40% similarity to any other gene, and phylogenetic analysis showed that STKV class formed a unique clade which is more related with drosophila *pelle *gene than plant species *STK *genes. The low similarity observed among STKV class with other four STK classes is not rare case for plant immunity genes. For example, resistance (*R*) gene, the other component of plant immune system, sometimes showed low sequence similarities. Resistance gene candidates (RGCs) sequence similarity ranged from 27 to 50% in *Zingiber *[28] and 19-44% in lettuce [29]. These data suggests that *STK *gene, as well as *R *genes, may exist in the genome long history and have diversified greatly during the long history evolution.

### Defense system in chestnut rose and its evolution

From a powdery mildew resistant genotype of chestnut rose, the component gene of immune system, i.e. *R*-like gene, kinase genes, *PR *genes, are all cloned [25,26,30]. In this study, we observed that STK2 and STK8 genes showed a close relationship with *Pto *Resistance gene, possibly indicated that they are candidate genes involved in the defense system in chestnut rose. When considering with the previous studies together, we found that for each plant immunity component gene many polymorphic members exist in the chestnut rose genome. This suggests that in the chestnut rose genome there is a sufficient resource of immunity genes utilized by plants to defend against pathogen attacks. Evolutionary analysis of these immunity genes also provided some clues on the complexity of plant immune system. Diversifying selection has been detected on RGC genes [25]; and for *PR2 *genes diversifying selection is predominant [30] in chestnut rose. The genes under diversifying selection are believed to have strong function in the defense, suggesting that in chestnut rose *PR2 *and *R *genes are actively involved in the immune responses which also require kinase gene for defense signaling. However, the immune system located in the plant cell is more complex than we can imagine. For example, there are more than 300 defensin-like genes in *Arabidopsis *[31]; many kinase genes such as Calmodulin-like domain protein kinases (CDPKs), and MAP kinases (MAPKs) were reported to be involved in defense activities [3,32]. Thus, plants can utilize enough strategies to defend against a pathogen invasion during the long time history.

## Conclusions

From a wild fruit crop 'chestnut rose', serine/threonine kinase (STK) genes were cloned by degenerate PCR. Fifteen STK sequences were isolated, and nine of them have continued to open reading frames, and they are separated into five classes based on sequence analysis. One of the classes (STK V) showed less than 40% similarity to any other new type of *STK *genes, and these new type STK genes exist as single copies in the genome, and they are phylogenetically related with animal homologs rather than plant homologs. The polymorphic STK genes, combined with other plant immunity genes, provide sufficient resources for the plants to defend against pathogen attacks.

## Competing interests

The authors declare that they have no competing interests.

## Authors' contributions

QX and XXD designed the research. QX carried out experiments. QX drafted the manuscript. XXD proposed and supervised the research. All authors read and approved the final manuscript.
